# Affiliated Fusion Conditional Random Field for Urban UAV Image Semantic Segmentation

**DOI:** 10.3390/s20040993

**Published:** 2020-02-12

**Authors:** Yingying Kong, Bowen Zhang, Biyuan Yan, Yanjuan Liu, Henry Leung, Xiangyang Peng

**Affiliations:** 1 Nanjing University of Aeronautics and Astronautics, Nanjing 210016, China; vinzhang@nuaa.edu.cn (B.Z.); yanbiyuan@nuaa.edu.cn (B.Y.); liuyanjuan@nuaa.edu.cn (Y.L.); 2 Nanjing Research Institute of Electronics Engineering, Nanjing 210007, China; wwukt@163.com; 3 University of Calgary, Calgary, AB T2P 2M5, Canada; Leungh@ucalgary.ca

**Keywords:** semantic image segmentation, CRF, DSM, UAV, remote sensing

## Abstract

Unmanned aerial vehicles (UAV) have had significant progress in the last decade, which is applied to many relevant fields because of the progress of aerial image processing and the convenience to explore areas that men cannot reach. Still, as the basis of further applications such as object tracking and terrain classification, semantic image segmentation is one of the most difficult challenges in the field of computer vision. In this paper, we propose a method for urban UAV images semantic segmentation, which utilizes the geographical information of the region of interest in the form of a digital surface model (DSM). We introduce an Affiliated Fusion Conditional Random Field (AF-CRF), which combines the information of visual pictures and DSM, and a multi-scale strategy with attention to improve the segmenting results. The experiments show that the proposed structure performs better than state-of-the-art networks in multiple metrics.

## 1. Introduction

Conditional Random Field (CRF) is a structured prediction model based on graph structures, which is a Markov Random Field (MRF) of random variables Y for given random variables X [1]. CRF has been widely used in various structured prediction tasks, such as estimating scores in Go games [2], separating specific genes from DNA [3], word segmentation in the field of natural language processing [4,5], and image segmentation for computer vision [6,7], etc. Since CRF can utilize lower-level contextual information at the structural level, it is well suited for these structured prediction tasks.

Semantic image segmentation, a pixel-level recognition task labeling the object category to each pixel in the image, is a typical structured prediction task. Semantic image segmentation is the basis of scene-parsing tasks, which have great potential in further applications such as automatic cruise, the landing of drones, and autonomous vehicles. The early application of CRF in the field of semantic image segmentation mainly utilizes a second-order CRF to model an image [8], which reads the associated information of four neighborhoods for each pixel, and uses two types of potential functions to model the conditional probability. The used potential functions are a unary potential function, which is only related to the current pixel feature, and a pairwise potential function, which is associated with pixels within the neighborhood. The precise inference of such methods often requires a large computational cost. Therefore, the maximum posterior estimation of the approximated conditional probability is usually obtained by sampling methods or variational methods.

The four-neighbor connectivity CRF has achieved a degree of success in certain fields, yet it also has inherent drawbacks in the model structure: four-neighbor communications can only consider the neighboring dependence of adjacent pixels, but not the spatial position. It cannot establish a long-range dependence on two pixels that are far away from each other in spatial position so that satisfactory segmentation results cannot be obtained when there is a large cross area, occlusion, repetition, or deformation in the category of the target. In order to solve this problem, a series of CRFs using high-order potential functions are proposed to improve their performance [9,10], which solve the above problems in certain aspects, but the computational complexity of the model is also significantly increased simultaneously.

To solve the above problems, a fully connected CRF with Gaussian kernels in the feature space is proposed [11], which has achieved a good compromise between speed and precision. However, CRFs that use specific functions are still not robust enough, resulting in complex textures and reduced performance, especially with changes in the proportion of long-range images such as those taken by drones or satellites. In order to better consider the spatial information of long-range pixels in order to reduce error classifications in the image parts with complex texture, we introduce geographical information through a digital surface model (DSM). To get a further improvement of segmentation accuracy, this paper proposes a fully connected Affiliated Fusion CRF (AF-CRF) with DSM, which (1) adopts a fully connected CRF structure to take long-range dependence into account; (2) utilizes the corresponding DSM to comprehend the height information of the region of interest (ROI); and (3) takes multi-scale pyramids for both images and DSM for global feature understanding. Compared to a large amount of manually labeled pictures, DSM can be generated automatically by the pictures taken above the ROI. The extra information of elevation of the interested region is believed to improve the performance of the classifier.

## 2. Preliminary

CRF is a discriminant undirected graph model. Compared with the corresponding generative model, it directly models the conditional probability. In this section, we simply introduce the theory of CRF and its general models.

### 2.1. Conditional Random Field

The idea of CRF is to model the conditional probabilities of multiple variables with given observations. Let $x=\left\{x_{1},x_{2},\cdots ,x_{n}\right\}$ be the observation sequence and $y=\left\{y_{1},y_{2},\cdots ,y_{n}\right\}$ be the corresponding label sequence. The purpose of CRF is to model the conditional probability P(y|x). ***y*** is a structural variable so that some relationship exists between its components.

Let G={V,E} denote undirected graphs with one-to-one correspondence between the observation sequence **x** and its label **y**. $y_{v}$ denotes an annotation variable corresponding to node *v*, and *n*(*v*) denotes adjacent nodes of *v*. If each variable $y_{v}$ of the graph satisfies the Markov property, then there is

(1) $$P\left(y_{v}|x,y_{V\backslash \left\{v\right\}}\right)=P\left(y_{v}|x,y_{n\left(v\right)}\right),$$

where V\{*v*} denotes all nodes except *v*.

### 2.2. General CRF Model

From the view of Equation (1), a CRF adds an observation sequence to a MRF, i.e., a CRF is a MRF given the observations. Similar to MRF, CRF defines the conditional probability distribution with potential functions and the cliques on the graph. According to Hammersley–Clifford theory, the probability distribution between multiple variables can be decomposed into a product of multiple factors based on the cliques in the graph, in which each factor is related only to a single maximum clique of the graph [12]. Specifically, for an observation sequence $x=\left\{x_{1},x_{2},\cdots ,x_{n}\right\}$ and its corresponding label sequence $y=\left\{y_{1},y_{2},\cdots ,y_{n}\right\}$, let *C* denote the set of maximum cliques and let $\left(x_{C},y_{C}\right)$ denote the variables corresponding to a single maximum clique C ∈ C; then, the conditional distribution *P* (**y**|**x**) can be written as

(2) $$P\left(y|x\right)=\frac{1}{Z}\prod_{C\in C}\Psi_{C}\left(y_{C},x_{C}\right),$$

where $Z=\sum \prod_{C\in C}\Psi_{C}\left(y_{C},x_{C}\right)$ is the partition function that normalizes $\prod_{C\in C}\Psi_{C}\left(y_{C},x_{C}\right)$.

In particular, a general CRF model is applied to the problem of semantic image segmentation. Consider a random field **X** defined on a set of variables $x=\left\{x_{1},x_{2},\cdots ,x_{n}\right\}$ with label y∈ *L* = {*l*_1_, *l*_2_ …, *l_k_*} and another random field **I** defined on another set of variables {*I*_1_, *I*_2_, …, *I_k_*}, in which **I** denotes an input image of size *N* and **X** denotes the pixel-level annotation of **I**. Then, the conditional probability distribution P(X|I) can be expressed as

(3) $$P\left(X|I\right)=\frac{1}{Z\left(I\right)}\exp(-\sum_{c\in C_{G}}\phi_{c}\left(X_{c}|I\right)),$$

where G=(V,E) is the graph defined on **X**. $C_{G}$ is the set of all the cliques in G and every clique *c* in $C_{G}$ introduces a potential function $\phi_{c}$ [13]. The Gibbs energy of the annotation $x\in L^{N}$ is

(4) $$E\left(x\right)=\sum_{c\in C_{G}}\phi_{c}\left(x_{c}|I\right).$$

Then, the MAP estimation of the CRF is

(5) $$x^{*}=\mathrm{argma}x_{x\in L^{N}}P\left(x|I\right).$$

In a fully connected CRF, let G be the complete graph of **X**, in which $C_{G}$ is the set of both unary cliques and pairwise cliques; then, the corresponding Gibbs energy is

(6) $$E\left(x\right)=\sum_{i}\phi_{u}\left(x_{i}|I\right)+\sum_{i<j}\phi_{p}\left(x_{i},x_{j}|I\right).$$

The unary potential $\phi_{u}\left(x_{i}|I\right)$ is only related to the feature of a single pixel itself, while the pairwise potential $\phi_{p}\left(x_{i},x_{j}|I\right)$ is related to the similarities and differences between every two pixels, which can be expressed as the linear combination of the Gaussian kernels defined in the feature space

(7) $$\phi_{p}\left(x_{i},x_{j}|I\right)=\mu \left(x_{i},x_{j}\right)\sum_{m=1}^{M}w^{\left(m\right)}\left(x_{i},x_{j}\right)k^{\left(m\right)}\left(f_{i}-f_{j}\right),$$

where $\mu \left(x_{i},x_{j}\right)$ is the label compatibility function to measure the probability that two labels appear at the same time. For instance, the probability of annotation {ship} and {waters} should be larger than one of {plane} and {waters}, intuitively.

## 3. Affiliated Fusion Conditional Random Fields

In this section, we describe the proposed model applying to semantically segment urban unmanned aerial vehicles (UAV) images. The first is a brief introduction to the theory of multi-scale and attention analysis. Afterwards, the Affiliated Fusion CRF is proposed.

### 3.1. Multi-Scale Analysis

Multi-scale analysis is a common method of digital image processing, which involves the representation and analysis of images at multiple resolutions. The advantages of this approach are obvious, in which features that cannot be detected at one resolution are often easier to be detected at another resolution. This section uses the classic image Gaussian pyramid as a multi-scale metric.

The image pyramid is a series of images of the pyramid structure obtained by the original image after multiple times of downsampling operation with the same ratio. The original image size and resolution of the bottom layer are the highest, and the resolution of the upper layer is reduced. The images in each layer have different sizes and resolutions. A complete image pyramid has *n* + 1 layers of image. The use of different scale representations of an image can be thought of as adding another dimension to the image. In addition to the conventional positional dimension (*x*,*y*), a dimension for depicting the current number of pyramid layers is added. This structure is also called the scale space.

According to the sampling theorem, only a fine structure that is sampled with less than 1/4 wavelength can be eliminated by a smoothing filter so that a correct downsampled image can be obtained. From a scale space perspective, this means that reducing the size of the image needs to be done in synchrony with the proper smoothing operation. The smoothing operation of the image can be performed by various low-pass filters, and the image pyramid obtained by Gaussian smoothing filter can be expressed as

(8) $$G\left(x,y\right)=\frac{1}{2\pi \sigma^{2}}exp\left(-\frac{{\left(x-x_{0}\right)}^{2}+{\left(y-y_{0}\right)}^{2}}{2\sigma^{2}}\right).$$

### 3.2. AF-CRF

The unary potential $\phi_{u}\left(x_{i}|I\right)$ is only related to the feature of a single pixel, while the pairwise potential $\phi_{p}\left(x_{i},x_{j}|I\right)$ is related to the similarities and differences between every two pixels. Therefore, to take advantage of the contextual information in the scale space, we optimize $\phi_{p}\left(x_{i},x_{j}|I\right)$ in the proposed AF-CRF, which can be expressed as below with the parameters of the model, ***θ***, and we hide the image **I** from Equation (7) for convenience:

(9) $$\phi_{p}\left(x_{i},x_{j}|\theta \right)=\sum_{m=1}^{M}\mu^{\left(m\right)}\left(x_{i},x_{j}|\theta \right)w^{\left(m\right)}\left(x_{i},x_{j}\right)k^{\left(m\right)}\left(f_{i}-f_{j}\right),$$

where $k^{\left(m\right)}\left(\cdot \right)$ refers to Gaussian kernel functions and *m* is the number of them. $k^{\left(m\right)}\left(\cdot \right)$ can be written as

(10) $$k^{\left(m\right)}\left(f_{i}-f_{j}\right)=\exp\left(-\frac{1}{2}{\left(f_{i}-f_{j}\right)}^{T}{\left(\Sigma^{-1}\right)}^{\left(m\right)}\left(f_{i}-f_{j}\right)\right),$$

where the vectors $f_{i}$ and $f_{j}$ are the feature vectors of pixel *i* and pixel *j* in the feature space, respectively. $w^{\left(m\right)}$ is the weight of each kernel, and $\mu^{\left(m\right)}\left(x_{i},x_{j}|\theta \right)$ is the label compatibility function. Each Gaussian kernel can be defined by the inverse of a covariance matrix ${\left(\Sigma^{-1}\right)}^{\left(m\right)}$.

The Gaussian kernels of the pairwise potential of the CRF with the DSM mentioned above are constructed with color vector ***I*,** position vector ***p***, and height *h*. The addition of *h* brings the spatial sensitivity in the pairwise potential of the model, which is defined in the Gaussian kernel as

(11) $$k^{\left(m\right)}\left(f_{i}-f_{j}\right)=w^{\left(1\right)}\exp\left(-\frac{{\left|p_{i}-p_{j}\right|}^{2}}{2\theta_{\alpha }^{2}}-\frac{{\left|I_{i}-I_{j}\right|}^{2}}{2\theta_{\beta }^{2}}-\frac{{\left|h_{i}-h_{j}\right|}^{2}}{2\theta_{\gamma }^{2}}\right)+w^{\left(2\right)}\exp\left(-\frac{{\left|p_{i}-p_{j}\right|}^{2}}{2\theta_{\delta }^{2}}\right),$$

where the first term considers that pixels with similar positions, similar colors and small height difference have higher probability of the same label categories. The second one considers the smoothing terms, which only points out that pixels with similar positions have a higher probability of the same label categories.

As for the label compatibility function $\mu^{\left(m\right)}\left(x_{i},x_{j}|\theta \right)$ that appeared in Equation (9), a simple and practical one is the Potts model, which can be written as

(12) $$\mu^{\left(m\right)}\left(x_{i},x_{j}|\theta \right)=I\left(l_{i},l_{j}\right),$$

where $I\left(l_{i},l_{j}\right)$ is a 0–1 indicator, which is

(13) $$I\left(l_{i},l_{j}\right)=\{\begin{array}{l} 1,l_{i}\neq l_{j}\\ 0,l_{i}=l_{j} \end{array}.$$

Although the segmentation result of utilizing the Potts model in CRF is generally acceptable, its disadvantage can be seen from its definition: the Potts model penalizes all inconsistent labels equally. For instance, the penalty of label{ship} and {waters} is equal to that of {plane} and {waters}, which is obviously not intuitive. In order to improve this simple Potts model, we can learn a symmetric adaptive category associated label compatibility function, which is

(14) $$\mu \left(l_{i},l_{j}|\theta \right)=\mu \left(l_{j},l_{i}|\theta \right).$$

Therefore, a more reasonable penalty is imposed on the inconsistency of the annotation in the semantic segmentation result of the UAV images.

Taking attention into account with inputting the images in *S* scales to the model and upsampling the output in all scales to the original size, the attention feature $f_{i,c}^{s}$ is the interest value of the *i*th pixel belongs to class *c* on the scale *s* with the pairwise potential of the CRF, where s∈{1,2,⋯,S}. Let $g_{i,c}$ denote the weighted sum of position *i* and class *c* of all the attention features of the representation, i.e., $g_{i,c}=\sum_{s}\delta_{i}^{s}f_{i,c}^{s}$, where the weight $\delta_{i}^{s}$ for each scale is

(15) $$\delta_{i}^{s}=e^{h_{i}^{s}}/\sum_{t=1}^{S}e^{h_{i}^{t}}.$$

$h_{i}^{t}$ is the output scores of each class (ranges from 1 to *C*) in each position (ranges from 1 to *N*). $\delta_{i}^{s}$ reflects the importance of position *i* in scale *s*, which is shared in all channels.

The attention term describes the relationship among the different scales of the CRF’s input and combines the differences of focused performance in the different scales with an adaptive weight. With the attention term, the model considers multi-scale information in the inference stage to get a more reasonable prediction.

**Figure 1:**
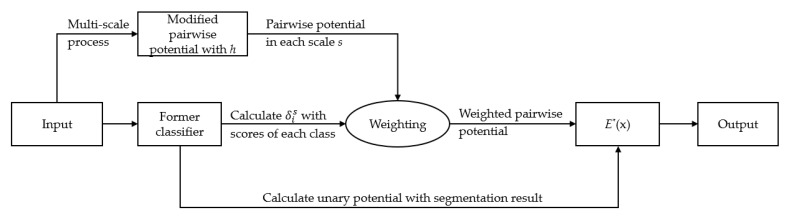
The workflow of Affiliated Fusion Conditional Random Field (AF-CRF).

Assume that the input scales are s∈{1,2,⋯,S} and combine the attention term proposed in the previous section. Considering the pairwise potential as an auxiliary decision condition in the inference stage, the Gibbs energy of the proposed model can be rewritten as

(16) $$E^{*}\left(x\right)=\sum_{i}\phi_{u}\left(x_{i}|I\right)+\sum_{s}\delta_{i}^{s}\sum_{i<j}\phi_{p,s}\left(x_{i},x_{j}|I\right).$$

where $\delta_{i}^{s}=e^{h_{i}^{s}}/\sum_{t=1}^{S}e^{h_{i}^{t}}$ as shown in Equation (15), and $\phi_{p,s}\left(\cdot \right)$ denotes the pairwise potential of the scale *s*. The new $E^{*}\left(x\right)$ considers the features of color, position, and height, as well as scale, which theoretically helps improve the robustness of the overall model. The workflow is illustrated in Figure 1.

## 4. Inference and Learning of the Model

In a fully connected CRF, the bottleneck of computing speed lies in the message-passing step. In order to achieve the fast operation of the proposed AF-CRF, this paper adopt the fast-solving algorithm with the mean field approximation proposed in [11] instead of the precise inference. The model inference is transformed into a Gaussian filtering in the feature space to improve the operation speed and achieve the acceptable accuracy. This section introduces the inference of conditional random fields and the learning methods of their parameters.

### 4.1. Inference

According to Equation (16), the exact probability distribution can be written as

(17) $$P\left(X\right)=\frac{1}{Z}\tilde{P}\left(X\right)=\frac{1}{Z}\exp\left(\sum_{i}\phi_{u}\left(x_{i}|I\right)+\sum_{s}\delta_{i}^{s}\sum_{i<j}\phi_{p,s}\left(x_{i},x_{j}|I\right)\right).$$

In order to achieve the fast operation of the proposed AF-CRF, the mean field approximation method is used in this section; that is, the probability distribution of the minimum Kullback-Leibler divergence (KL divergence) between the calculation and the accurate probability distribution is approximated instead of calculating the exact probability distribution directly. The approximate probability distribution can be expressed as the product of a series of marginal probability distributions as

(18) $$Q\left(X\right)=\prod_{i}Q_{i}\left(X_{i}\right),$$

where $Q_{i}\left(X_{i}\right)$ is the marginal distribution of each variable in the model. Then, the KL divergence of Q(X) and P(X) can be expressed as

(19) $$\begin{array}{ll} D\left(Q||P\right) & =\sum_{x}Q\left(x\right)\log\frac{Q\left(x\right)}{P\left(x\right)}\\ & =-\sum_{x}Q\left(x\right)\log P\left(x\right)+\sum_{x}Q\left(x\right)\log Q\left(x\right)\\ & =-E_{U∼Q}\left[\log P\left(U\right)\right]+E_{U∼Q}\left[\log Q\left(U\right)\right]\\ & =-E_{U∼Q}\left[\log\tilde{P}\left(U\right)\right]+E_{U∼Q}\left[\log Z\right]+\sum_{i}E_{U_{i}∼Q}\left[\log Q\left(U_{i}\right)\right]\\ & =E_{U∼Q}\left[E\left(U\right)\right]+\sum_{i}E_{U_{i}∼Q_{i}}\left[\log Q_{i}\left(U_{i}\right)\right]+\log Z, \end{array}$$

where $E_{U∼Q}$ denotes the expectation in the distribution *Q*. Q(x) can be derived as a product of independent marginal distribution, which is $Q\left(X\right)=\prod_{i}Q_{i}\left(X_{i}\right)$. In this paper, the Q(X) of each scale is inferenced separately to focus on the specific scale, which is denoted as $Q_{s}\left(X\right)$. In order to minimize the KL divergence while ensuring that $Q_{s}\left(X\right)$ and $Q_{s,i}\left(X_{i}\right)$ consist of the proper probability density (i.e., $\int Q_{s}\left(X\right)=1$ and $\int Q_{s,i}\left(X_{i}\right)=1$), the following iterative updating formula is used:

(20) $$Q_{s,i}\left(x_{i}=l\right)=\frac{1}{Z_{i}}\exp\left(-\phi_{u}\left(x_{i}\right)-\sum_{l^{\prime }\in ℒ}\mu \left(l,l^{\prime }\right)\sum_{m=1}^{K}w^{\left(m\right)}\sum_{j¹i}k^{\left(m\right)}\left(f_{i}-f_{j}\right)Q_{s,j}\left(l^{\prime }\right)\right).$$

From the view of signal processing, kernel functions can simplify inner product operation in a mapping space. Equation (20) can be expressed as the convolution of Gaussian kernels in feature space, which is

(21) $${\tilde{Q}}_{s,i}^{\left(m\right)}\left(l\right)=\sum_{j\in V}k^{\left(m\right)}\left(f_{i},f_{j}\right)Q_{s,j}\left(l\right)-Q_{s,i}(l)=\left(G_{{\left(\Sigma^{-1}\right)}^{\left(m\right)}}⊗Q_{s}(l)\right)(f_{i})-Q_{s,i}(l),$$

where $G_{{\left(\Sigma^{-1}\right)}^{\left(m\right)}}$ refers to the Gaussian kernel defined by ${\left(\Sigma^{-1}\right)}^{\left(m\right)}$. The convolution is represented as a low-pass filter. According to the sampling theorem, the function can be reconstructed from a set of samples whose spacing is proportional to the standard deviation of the filter [11]. Therefore, we can perform convolution by downsampling $Q_{s}\left(l\right)$, using $G_{{\left(\Sigma^{-1}\right)}^{\left(m\right)}}$ for the convolution, and upsampling the results at the feature points [14].

Truncated Gaussian approximation is a common approximation for Gaussian kernels, in which all values exceeding two standard deviations are set to zero. Since the spacing of samples is proportional to the standard deviation, the truncated Gaussian kernels only support a constant number of sample points. Therefore, by aggregating values from only a constant number of adjacent samples, the convolution can be approximately calculated at each sample.

### 4.2. Learning

The data-driven parameters that need to be learned are the symmetric label compatibility functions mentioned in previous sections. The symmetry of *μ* is beneficial to realize the learning algorithm on the one hand and also to the intuition on the other hand: the punishment of the label {waters} for {ship} should be equal to the penalty of the label {ship} for {waters}. In order to effectively learn this symmetric label compatibility function *μ*, this paper uses the maximum likelihood estimation (MLE) criterion. The goal of MLE is to find a set of parameters that maximize the log-likelihood of the model given the training image *I* and its annotation result *T* ^(*n*)^, which is

(22) $$\begin{array}{ll} l\left(\mu :T^{\left(n\right)},I^{\left(n\right)}\right) & =\log P\left(X=T^{\left(n\right)}|I^{\left(n\right)},\mu \right)\\ & =-E\left(T^{\left(n\right)}|I^{\left(n\right)},\mu \right)-logZ\left(I^{\left(n\right)},\mu \right), \end{array}$$

where $I^{\left(n\right)}$ is the training image and $T^{\left(n\right)}$ is its annotation.

The Limited-memory Broyden-Fletcher-Goldfarb-Shanno (L-BFGS) algorithm is adopted to learn the label compatibility functions to maximize the log-likelihood $l\left(\mu :T^{\left(n\right)},I^{\left(n\right)}\right)$. L-BFGS requires gradients to be calculated, which is difficult to estimate accurately because of the calculation of the gradient of the normalization factor *Z*. Therefore, the mean field approximation described in the previous section is used to estimate the gradient of *Z*. That is, a simple approximation of the gradient of each training image, which is

(23) $$\frac{\partial }{\partial \mu \left(a,b\right)}l\left(\mu :T^{\left(n\right)},I^{\left(n\right)}\right)\approx -\sum_{i}T_{i}^{\left(n\right)}\left(a\right)\sum_{j<i}k\left(f_{i}-f_{j}\right)T_{i}^{\left(n\right)}\left(b\right)+\sum_{i}Q_{i}\left(a\right)\sum_{j<i}k\left(f_{i}-f_{j}\right)Q_{i}\left(b\right),$$

where $T_{i}^{\left(n\right)}\left(a\right)$ is a 0–1 indicator. $T_{i}^{\left(n\right)}\left(a\right)=1$ when the annotation of $I^{\left(n\right)}$’s *i*th pixel is *a*, i.e., $T_{i}^{\left(n\right)}\left(a\right)=a$ and $T_{i}^{\left(n\right)}\left(a\right)=0$ otherwise.

## 5. Experiments and Analysis

In this section, we first introduce the dataset we use and the metric utilized to test the model. Afterwards, some experiments are conducted to test the proposed AF-CRF. The experiments demonstrate that the proposed method increases both the global accuracy (GA) and the Intersection over Union (IoU) and obtains a state-of-the-art result.

### 5.1. Dataset

**Figure 2:**
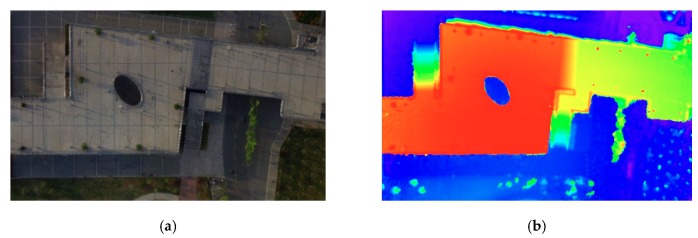
The unmanned aerial vehicles (UAV) image and the corresponding digital surface model (DSM) used in the experiment. (**a**) The UAV image; (**b**) the corresponding DSM.

**Figure 3:**
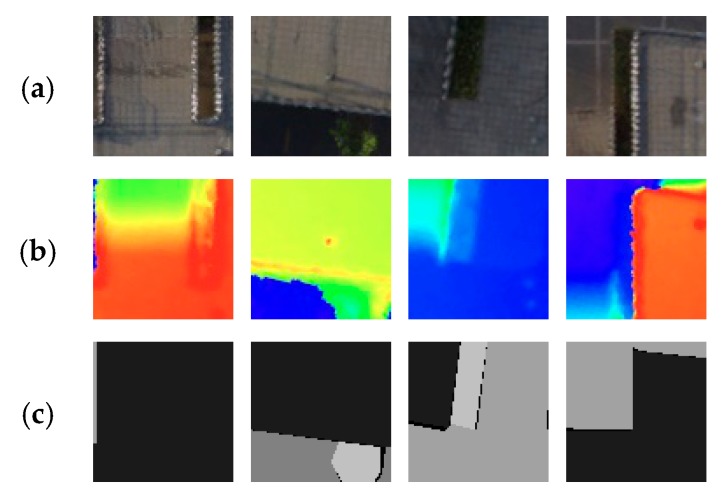
The 256 × 256 slices. (**a**) UAV cut images; (**b**) The corresponding DSM; (**c**) Ground Truth.

The UAV urban images are taken by DJI Phantom 3 Standard at a height of 40 m, and the DSM is generated by PhotoScan [15] at a resolution of 2560 × 1536. The image for experiments is resized to the same resolution so that the DSM is manually adjusted to the image with only translation and rotation. Then, we cut the original image and DSM randomly to 160 pieces of 256 × 256 slices (60 pieces in order without overlapping and 100 pieces at random with overlapping) to train the networks. The image is labeled into five categories: background (not counted in the result), bridge (cls1), road (cls2), sidewalk (cls3), and vegetation (cls4). We randomly select 140 pairs to train and 20 pairs to validate. The origin image, DSM, and the cut slices are shown in Figure 2 and Figure 3, respectively.

### 5.2. Implement Details

The model is trained with L-BFGS as described above and the loss is per-pixel softmax, which is a multinomial cross-entropy loss in terms of the predicted label *x* and the ground truth *y*, which is

(24) $$loss(x,y)=-\log\frac{\exp(x_{cls})}{\sum {}_{j}\exp(x_{j})}.$$

Here, $x_{\mathrm{cls}}$ is the predicted score of the ground truth class *y*.

The unary potential adopts the output of some previous works for semantic image segmentation, such as PSPNet-*ds-ss* [16], and sets it as a priori probability that the output is correct, which in this paper is set to 0.5 with a negative logarithm operation. The output score $h_{i}^{t}$ in Equation (15) adopts the output of the last layer of PSPNet-*ds-ss*. The label compatibility function is initialized with the Potts model, which performs an identity matrix in the first training stage.

### 5.3. Results and Discussion

To get a quantitative evaluation result, we adopt global accuracy (GA) and intersection over union (IoU) as metrics, which are

(25) $$GA=\sum_{i=1}^{k}n_{ii}/\sum_{i=1}^{k}t_{i},$$

(26) $$IoU_{\mathrm{cls}}=\frac{n_{ii}}{t_{i}-n_{ii}+\sum_{j=1}^{k}n_{ji}},$$

where *t_i_* is the total number of pixels of class *i* and the subscript cls means the accuracy within the specific class. *k* is the number of classes and *n_ij_* is the number of pixels that belong to class I and were classified to class *j*. GA represents the performance of the training, while IoU penalizes the false positive classification to demonstrate the performance in semantic segmentation. To get a general evaluation, mean intersection over union (mIoU) is also adopted, which is

(27) $$\mathrm{mIoU}=\frac{1}{k}\sum_{i=1}^{k}\frac{n_{ii}}{t_{i}-n_{ii}+\sum_{j=1}^{k}n_{ji}}.$$

Aiming at further applications of semantic segmentation, we also adopted a metric of confidence for the model’s outputs, which indicates the probability of the final output category. The confidence of the outputs matters when decisions are made by multi-sensor fusion.

**Figure 4:**
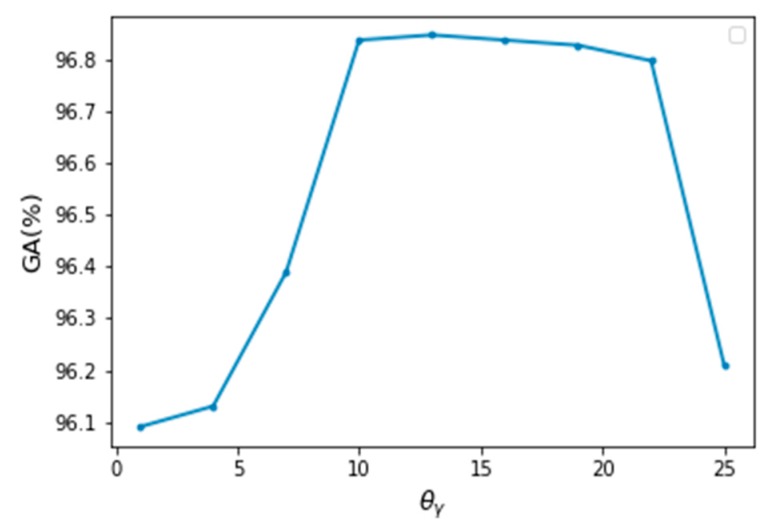
Global accuracy varies with the change of $\theta_{\gamma }$.

According to Equations (12) and (16), the main parameters of the model are $\theta =\left\{\theta_{\alpha },\theta_{\beta },\theta_{\gamma },\theta_{\delta }\right\}$, $w=\left\{w^{\left(1\right)},w^{\left(2\right)}\right\}$, and the label compatibility function μ(⋅). Due to the model settings, the value of $w=\left\{w^{\left(1\right)},w^{\left(2\right)}\right\}$ is always near 1 (generally 1 + 0.001). The smoothing parameters of the position weight has little effect on the experimental results, so it is set here as a fixed value $\theta_{\delta }$ = $w^{\left(1\right)}$ = $w^{\left(2\right)}$ = 1. According to [11], this paper also set $\theta_{\alpha },\theta_{\beta }$ = 13 here. With regard to the height weight parameter $\theta_{\gamma }$, when $\theta_{\gamma }$ = $\theta_{\alpha }$ = $\theta_{\beta }$ = 13 by grid searching, the model has the best performance, as shown in Figure 4.

.

**Figure 5:**
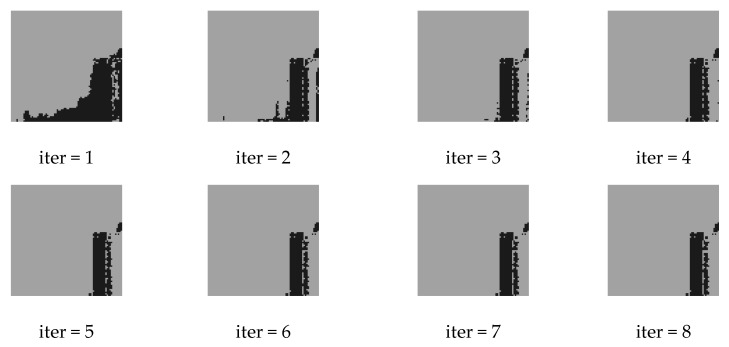
Outputs of the origin CRF model in every inference iteration, which is nearly converged within five iterations.

**Figure 6:**
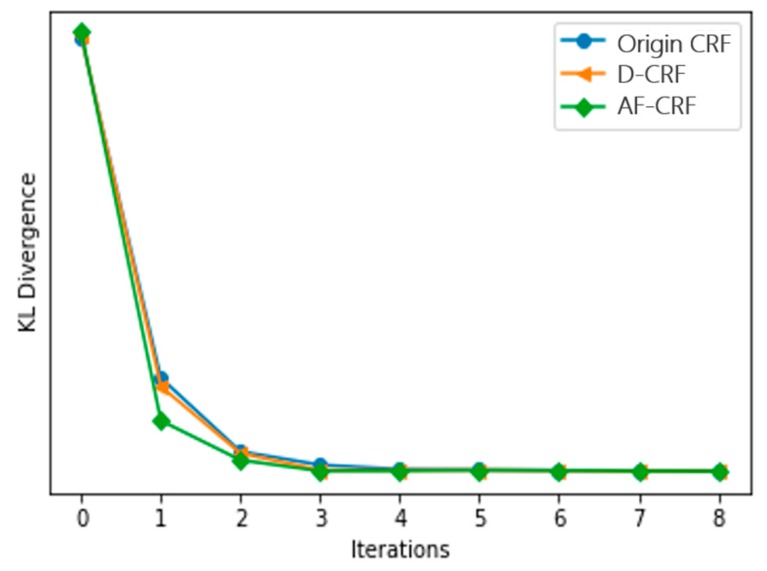
Comparison of KL divergence convergence of three CRF models.

Figure 5 and Figure 6 are the results of eight iterations of the original CRF model and the convergence of KL divergence of the three conditional random fields, respectively, where D-CRF refers to a Dual-CRF (images and DSM) without the multi-scale strategy. As demonstrated, the KL divergence of the models is generally convergent within five iterations; hence, in subsequent experiments, the number of iterations was set to five. In addition, Figure 6 also reveals that the proposed AF-CRF has the fastest convergence speed among the three kinds of CRF.

**Figure 7:**
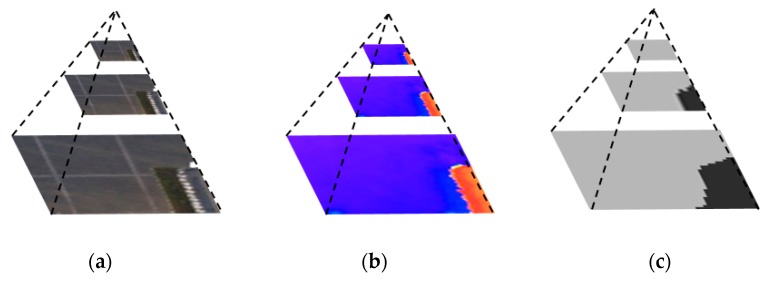
Multi-scale pyramid demonstration. (**a**) Original image pyramid; (**b**) Corresponding DSM pyramid; (**c**) Output pyramid.

**Figure 8:**
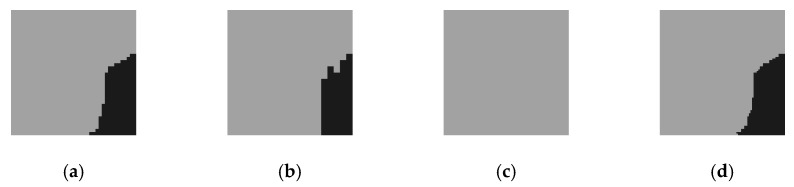
Multi-scale outputs. (**a**) Scale 1/2; (**b**) Scale 1/4; (**c**) Scale 1/8; (**d**) Synthesis results based on attention.

Figure 7 is the pyramid structure of image–DSM–output. The higher the pyramid layers, the simpler the original image and the features of the DSM, and the more concentrated the attention of the output results. The output of the three scales is shown in Figure 8. When the scale factor is 1/8, the attention range is reduced to the extreme, and the output of the whole image is of the same category. On the one hand, it reflects the characteristics of attention in different scales. On the other hand, it also indicates that in practicality, the pyramid scale should not be too large; otherwise, it will lose the relevant features of the region of interest. In subsequent experiments, the number of pyramid scales was only two, i.e., the scale of 1/2 and the scale of 1/4.

**Figure 9:**
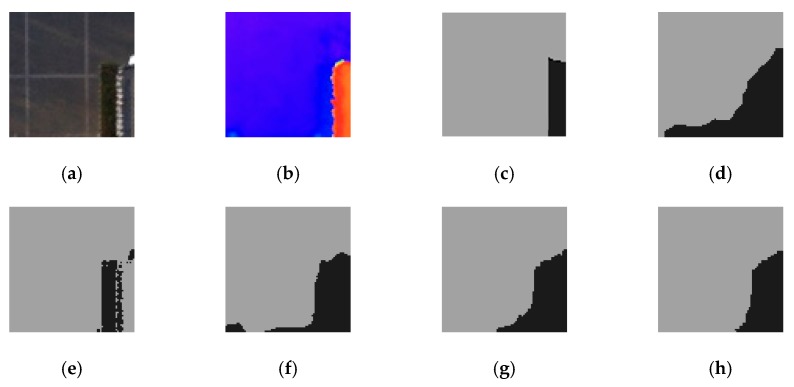
Comparison of the input with output. (**a**) Original image; (**b**) Corresponding DSM; (**c**) Ground truth; (**d**) Fully Convolutional Network (FCN) output; (**e**) Original CRF output (with picture input); (**f**) Original CRF output (with DSM input); (**g**) Dual-CRF (D-CRF) output; (**h**) AF-CRF output.

The overall performance of the proposed AF-CRF is shown in Figure 9. Compared with the output of other models, the output of AF-CRF is the closest to the ground truth, while carefully retaining the output characteristics of the Fully Convolutional Network (FCN) classifier. A quantitative evaluation is shown in Table 1. Since the evaluation of PSPNet*-ds-ss* on several quantitative evaluations has reached a fairly high level, the performance of AF-CRF has only slightly improved. In order to evaluate the performance of AF-CRF better and fairly, a W-5 (Worst-5) index is proposed, which is the improvement of the five worst results of data output after the model, as shown in Table 2. The proposed AF-CRF has significantly improved the disadvantage of the former classifier. 

**Figure 10:**
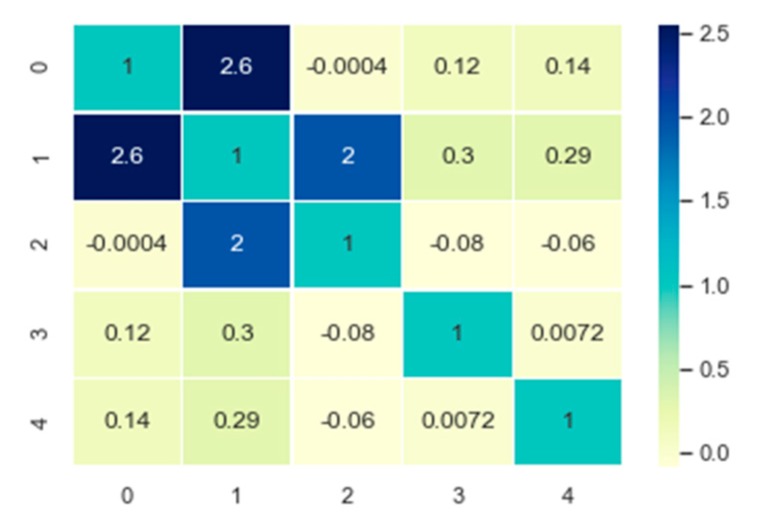
The value of the learned label compatibility parameters.

The values of the symmetric label compatibility function of the learned AF-CRF are shown in Figure 10, where the labels are {0: void}, {1: bridge}, {2: road}, {3: sidewalk}, and {4: vegetation}. From Figure 9, the result of learning is that the compatibility between label bridges and roads, as well as roads and sidewalks, is relatively large, while it restrains the label compatibility of roads and pavements and roads and vegetation, which is consistent with the dataset. On the other hand, the imbalance in datasets also affects the learning of label compatibility function, making it more focused on the category of {1: bridge} with the largest number.

We also test the improvements to some other networks, as shown in Table 3. FCN8s, DeepLab, and U-Net are all classical networks in the field of semantics image segmentation. Their primary output results have been significantly improved after AF-CRF post-processing.

**Figure 11:**
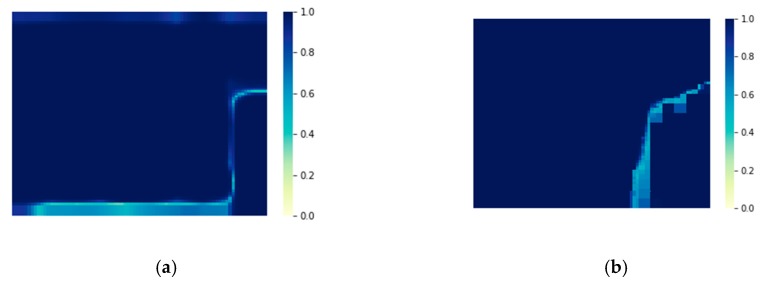
The confidence of model output. (**a**) PSPNet*-ds*-*ss*; (**b**) AF-CRF.

**Figure 12:**
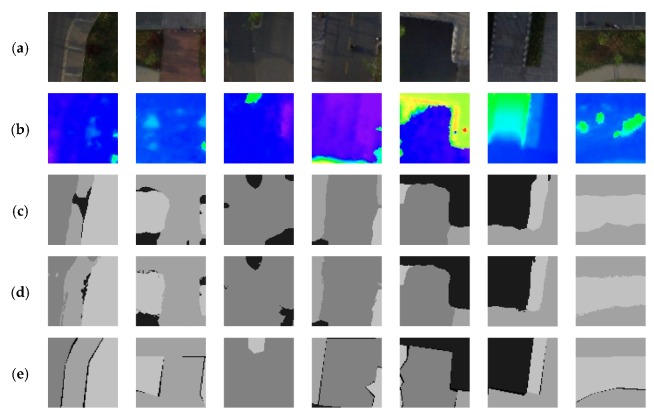
Examples of AF-CRF output. (**a**) UAV images; (**b**) Corresponding DSM; (**c**) Former classifier outputs; (**d**) AF-CRF outputs; (**e**) Ground Truth.

While focusing on the global accuracy and IoU, this paper also considers the confidence of the outputs of the model. As shown in Figure 11, the confidence of the model output is expressed as a thermogram of the probability of the classification results for each pixel in the image. It can be seen from the figure that the output of AF-CRF not only improves the accuracy of PSPNet-*ds-ss*, but also improves the output confidence of the former FCN classifier, especially in the edge-independent region. Due to the dense label–label connection of the AF-CRF model, the confidence of the outputs in the continuous area of large labeling (as shown in the upper and lower left of Figure 11) has been significantly improved. Confidence has a positive impact on the subsequent application of semantic UAV image segmentation, which to some extent affects the correct decision of subsequent multi-sensor fusion. The quantitative evaluation of the confidence index is shown in Table 4. AF-CRF is better than the PSPNet-*ds*-*ss* model in both the average confidence and minimum confidence of outputs. More examples of AF-CRF output are shown in Figure 12.

The proposed AF-CRF also has some drawbacks. As shown in the 3rd column of Figure 12, the wrong classification at the top of the image is difficult to correct. Due to the dense connection between annotations and the influence of the former classifier as a unary potential, AF-CRF inevitably neglects the correct categories that are not appearing in the output of the former one. In addition, as seen in the first column of Figure 12, after AF-CRF, the correct part of the output in the former classifier appears in the wrong classification spots, which is the result of an over-consideration of long-range dependence, and the categories in the location where the errors occur are all so flat that the corresponding DSM fails to provide additional information as well.

## 6. Conclusions

In this paper, we propose a novel Affiliated Fusion Conditional Random Field (AF-CRF) to semantically segment urban UAV images. The model adopts DSM as an extra geographical information to improve the segmentation result along with a multi-scale analysis with attention. The experiments show that our proposed model improves not only the accuracy but also the confidence of output results, which has considerable benefit to the further applications of urban UAV image semantic segmentation. The limitation of our method is that the DSM of regions of interest must be generated in advance, yet it will be easier to get as the geographic information system develops. The future work will focus on a more specified form of the unary potential.

## Figures and Tables

**Table 1:** The quantitative evaluation of AF-CRF and some other state-of-the-art models. GA: global accuracy, IoU: Intersection over Union.

Model	GA_T_ (%)	GA_V_ (%)	IoU_cls1_ (%)	IoU _cls2_ (%)	IoU _cls3_ (%)	IoU _cls4_ (%)	mIoU (%)
DeepLabv3 + *-ds-ss* [17]	94.62	91.55	79.36	57.36	65.16	59.76	63.16
PSPNet*-ds-ss*	97.79	96.83	92.67	74.90	81.12	72.14	80.27
DeepLabv3 + *-ds-ss* + CRF	95.57	93.29	85.45	57.33	69.97	62.31	65.87
PSPNet*-ds-ss +* CRF	97.75	96.85	93.21	74.71	83.26	72.14	81.02

**Table 2:** The W-5 index of AF-CRF and some other state-of-the-art models.

Model	GA (%)	IoU_cls1_ (%)	IoU _cls2_ (%)	IoU _cls3_ (%)	IoU _cls4_ (%)	mIoU (%)
DeepLabv3 + *-ds-ss*	72.37	71.24	43.20	54.81	42.55	53.24
PSPNet*-ds-ss*	77.28	80.67	61.90	69.14	53.39	67.77
DeepLabv3 + *-ds-ss+*CRF	85.34	81.47	54.27	60.72	60.55	71.09
PSPNet*-ds-ss +* CRF	90.97	90.12	70.01	78.21	69.85	78.82

**Table 3:** The improvements to other networks.

Model	CRF	GA (%)
FCN8s [18]	×	80.3
	√	85.4
DeepLab	×	78.2
	√	82.5
U-Net [19]	×	67.5
	√	79.3

**Table 4:** The confidence of the model in the outputs.

Metric	PSPNet-*ds-ss*	AF-CRF
Aver. Conf (%)	97.09	98.65
Min. Conf (%)	35.57	50.04
